# Function and regulation of Rnd proteins in cortical projection neuron migration

**DOI:** 10.3389/fnins.2015.00019

**Published:** 2015-02-06

**Authors:** Roberta Azzarelli, François Guillemot, Emilie Pacary

**Affiliations:** ^1^Cambridge Department of Oncology, Hutchison/MRC Research Centre, University of CambridgeCambridge, UK; ^2^Division of Molecular Neurobiology, MRC National Institute for Medical ResearchLondon, UK; ^3^Institut National de la Santé et de la Recherche Médicale U862, Neurocentre MagendieBordeaux, France; ^4^Université de BordeauxBordeaux, France

**Keywords:** Rho GTPases, Rnd, cortical development, neuronal migration, Plexin

## Abstract

The mammalian cerebral cortex contains a high variety of neuronal subtypes that acquire precise spatial locations and form long or short-range connections to establish functional neuronal circuits. During embryonic development, cortical projection neurons are generated in the areas lining the lateral ventricles and they subsequently undergo radial migration to reach the position of their final maturation within the cortical plate. The control of the neuroblast migratory behavior and the coordination of the migration process with other neurogenic events such as cell cycle exit, differentiation and final maturation are crucial to normal brain development. Among the key regulators of cortical neuron migration, the small GTP binding proteins of the Rho family and the atypical Rnd members play important roles in integrating intracellular signaling pathways into changes in cytoskeletal dynamics and motility behavior. Here we review the role of Rnd proteins during cortical neuronal migration and we discuss both the upstream mechanisms that regulate Rnd protein activity and the downstream molecular pathways that mediate Rnd effects on cell cytoskeleton.

## Introduction

During the development of the central nervous system, neural progenitors undergo a sequence of distinct cellular events to give rise to the vast array of neurons that populate the entire brain. In the cerebral cortex, excitatory projection neurons, which constitute the majority of cortical neurons, are generated from neural stem/progenitor cells located in the areas lining the lateral ventricles, the ventricular (VZ) and subventricular zones (SVZ) of the dorsal telencephalon (Franco and Muller, 2013; Marin and Muller, 2014). Soon after birth, young neuroblasts leave the proliferative areas and migrate to the cortical plate (CP), where they distribute into six horizontal layers and they establish local and long-range connections (Rakic, 1988; Nadarajah and Parnavelas, 2002; Martynoga et al., 2012; Greig et al., 2013). It is now increasingly evident that a highly motile cellular behavior is crucial for different aspects of cortical neurogenesis, including, but not restricted to radial migration of post-mitotic neurons. Indeed, progenitor cells in the VZ also exhibit motile characteristics, such as the migration of their nuclei in coordination with cell cycle progression.

The sequential steps of neurogenesis and migration are promoted by the extensive and dynamic remodeling of the cell cytoskeleton. It is indeed the rapid re-organization of the actin filaments and microtubule network that ultimately regulates the motility behavior of nuclei in cycling progenitors and of migrating neurons (Lambrechts et al., 2004; Heng et al., 2010; Taverna and Huttner, 2010). The importance of the control of cytoskeletal remodeling for cortical neurogenesis is highlighted by the fact that most of the genes mutated in human patients with cortical malformations produce cytoskeletal proteins or their regulators (Guerrini and Parrini, 2010; Friocourt et al., 2011).

Members of the Rho family of small GTPases are key regulators of cell cytoskeleton in various cell types (Ridley, 2001). Rho proteins act as molecular switches capable of fast cycles of activation and inactivation, which represent an ideal system to regulate the dynamic changes of the cytoskeleton during migration. Also, the spatial and temporal control over Rho GTPase activity within the cell enables differential regulation of cytoskeletal components in distinct subcellular compartments, driving for example protrusion formation at the front of a migrating cell and cell retraction at the rear. The Rho family includes not only the classical members, which cycle between an active GTP-bound state and an inactive GDP-bound state, but it also contains “atypical” members like the Rnd subfamily, which possess low or no intrinsic GTPase activity and are therefore considered to be constitutively active (Nobes et al., 1998; Chardin, 2006; Riou et al., 2010). Since Rnd proteins do not undergo the classical GTPase cycle, gene expression, protein post-transcriptional modifications and subcellular localization are predominant mechanisms that control Rnd activity. Interestingly, Rnd proteins evolved relatively recently and they are present only in vertebrates, indicating that they might be involved in more specialized neuronal functions than the other Rho GTPases (Chardin, 2006; Boureux et al., 2007). The role of Rnd proteins in cortical development has become subject of intensive research only recently. Here we review the functions and regulation of Rnd small GTPases during progenitor nuclear migration and during radial migration of cortical neurons.

## Neuronal migration in the cerebral cortex

### Interkinetic nuclear migration of neural progenitors

**Figure 1:**
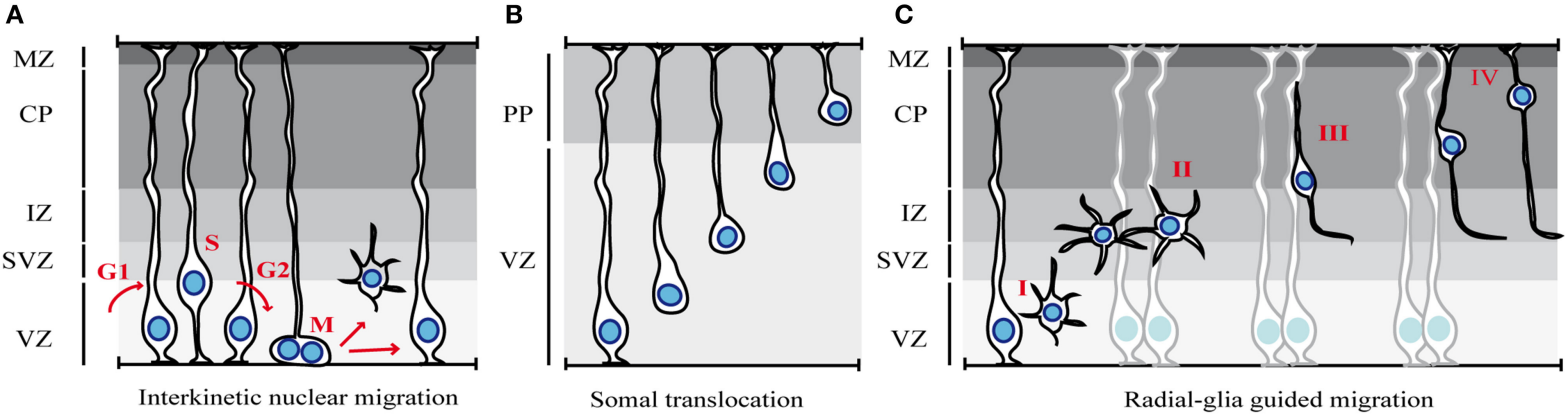
**Modes of migration in the cortex. (A)** Interkinetic nuclear migration. The nuclei of neuroepithelial cells or radial glia cells occupy different positions along the apical-basal axis depending on the phase of the cell cycle (see text for details). **(B)** Somal translocation of early-born cortical neurons. Newborn neurons lose their apical attachment and reach the PP by translocation of the soma and progressive shortening of the basal process. **(C)** Glia-guided radial migration of cortical neurons. Four phases of radial migration can be distinguished. Newborn neurons leave the proliferative areas **(I)** and reach the SVZ/IZ, where they acquire a multipolar morphology **(II)**. After pausing in the SVZ/IZ, cells migrate toward the CP, using locomotion **(III)**. At the end of their migration, cortical neurons switch to soma translocation (**IV**). MZ, marginal zone; CP, cortical plate; PP, preplate; IZ, intermediate zone; SVZ, subventricular zone; VZ, ventricular zone.

After closure of the neural tube, the epithelium lining the ventricles becomes a specialized neuroepithelium that consists of a single sheet of progenitors called neuroepithelial cells. At the onset of neurogenesis (~E10 in mouse), these cells self-renew to expand the progenitor pool and then convert into cells with glia-like features, the radial glial cells. A typical feature of these two populations of progenitors is the apico-basal movement of their nuclei in coordination with cell cycle progression, a phenomenon known as interkinetic nuclear migration (INM) (Sauer and Walker, 1959) (Figure 1A). In neuroepithelial cells, INM spans the entire apico–basal axis of the cell whereas in radial glia cells, this behavior is confined to the portion of the cell in the VZ. During G1 phase of the cell cycle, the nuclei of neural progenitor cells migrate from the apical to the basal side, where DNA replication occurs, whereas during G2 phase of the cell cycle, the nucleus moves toward the ventricular surface and undergoes mitosis at the most apical side. Since neural progenitors are not synchronized in their cell cycle and as a consequence of INM, the nuclei are found scattered in different apico-basal positions and the single layered neuroepithelium and the VZ appear pseudo-stratified. Interestingly, several lines of evidence indicate that INM is not required for cell cycle progression, whereas alteration of cell-cycle parameters may interfere with INM (Taverna and Huttner, 2010).

Although the contribution of INM to cortical neurogenesis has not yet been fully understood, it is possible that INM may allow packing more progenitor cells within a limited surface in order to maximize the mitoses of progenitor cells. Alternatively, INM may regulate progenitor fate by controlling the exposure of progenitor nuclei to proliferative vs. neurogenic signals (Taverna and Huttner, 2010; Spear and Erickson, 2012).

The translocation of the nucleus during INM requires dynamic changes of the cell cytoskeleton, with both actin and microtubule (MT) networks involved in this process (Taverna and Huttner, 2010). The relative contribution of MT- and actin-dependent mechanisms depends on the model organism and on the brain region investigated (Lee and Norden, 2013). In the developing rodent cortex, the basal-to-apical nuclear migration involves MT-based motors, whereas the apical-to-basal migration seems to depend on both actomyosin and MT-based motors (Schenk et al., 2009; Tsai et al., 2010). In addition, recent work proposes that the regulation of apical-to-basal nuclear migration during G1 is not an active, cell-autonomous process, but it involves a passive component (Kosodo et al., 2011). Kosodo and colleagues suggested that the basal nuclear movement during G1 is indirectly driven by the opposite movement of G2-phase nuclei migrating apically (Kosodo et al., 2011). Thus, the mechanisms underlying basal-to apical and apical-to-basal INM seem to exhibit profound differences.

### Radial migration of projection neurons

The majority of cortical neurons are excitatory glutamatergic cells that extend long projections toward cerebral and subcerebral targets. The first cohort of cortical neurons that migrate out from the VZ determine the formation of the preplate, a primitive structure that becomes soon divided into the superficial marginal zone and the deeper subplate by a subsequent wave of migrating neurons (Luskin et al., 1988). At the stage of preplate formation and early born neuron production, between E12 and E14 in the mouse, the main mode of migration is somal translocation (Miyata et al., 2001; Nadarajah et al., 2001) (Figure 1B). The cells that undergo somal translocation are born from radial glia cells at early developmental stages and they possess both apical attachment and basal radial process at the time of birth. After detachment form the ventricular surface, the continuous advancement of the nucleus and the concomitant retraction of the basal process determine a fast migratory behavior. Early-born neurons eventually occupy deep cortical layers since later-born neurons migrate and pass earlier born cells in order to settle progressively in upper cortical layers in an “inside-out” fashion.

At later developmental stages, after E14, when the cortical wall progressively increases in its thickness, neurons predominantly use a mode of radial migration called glia-guided migration (Rakic, 1972; Noctor et al., 2001) (Figure 1C). In contrast to somal translocation that is independent from radial glia fibers, glia-guided migration strictly relies on the radial glia scaffold. Young neurons that use this mode of migration lose contact with both the ventricle and the basal lamina and “embraced” the radial fiber during their migration. Newborn neurons usually migrate along the fiber of their mother radial glia, although they can jump from one fiber to another during migration, a process that regulates intermixing of neuronal clones within the cortex.

This entire process of radial migration can be subdivided into distinct phases (Figure 1C) (Nadarajah and Parnavelas, 2002; Noctor et al., 2004), in which neurons undergo rapid changes in cell polarity, morphology and speed of migration, as they progress from the VZ to the CP. The first step is characterized by the detachment of cells from the apical/ventricular surface in order to leave the proliferative zones and reach the SVZ and the intermediate zone (IZ) (Figure 1C-I). Then, post-mitotic neurons pause for a variable amount of time in the SVZ/IZ (maximum time recorded of 24 h), where they acquire a multipolar shape (Figure 1C-II). In this phase, neurons actively extend and retract dynamic processes, but they do not move significantly (Tabata and Nakajima, 2003). After sojourning in the IZ, neurons enter the CP. However, some neurons take a path toward the VZ, before reversing their direction of migration toward the CP (Noctor et al., 2004). The purpose of this migratory behavior is poorly understood. Once in the CP, neurons become bipolar, extending a leading process toward the pial surface and a trailing process in the direction of the IZ (Figure 1C-III), and migrate toward the upper layer of the CP. During this phase, nascent neurons use glia-guided migration (also called glia-guided locomotion), which is characterized by repetitive migratory cycles of extension of the leading process, translocation of the nucleus, and retraction of the trailing process. However, since the trailing process of migrating cortical neurons will become the future axon, it has been proposed that neurons do not retract the trailing process at the end of each migratory cycle, but rather extend their axon as they move (Noctor et al., 2004; Tabata et al., 2009; Hatanaka and Yamauchi, 2013). Finally, when projection neurons reach their destination, they undergo a last nuclear translocation without leading process extension, indicating that locomoting cells switch to somal translocation at the end of their migration (Nadarajah et al., 2001) (Figure 1C-IV).

## Classical and atypical Rho GTPases

**Figure 2:**
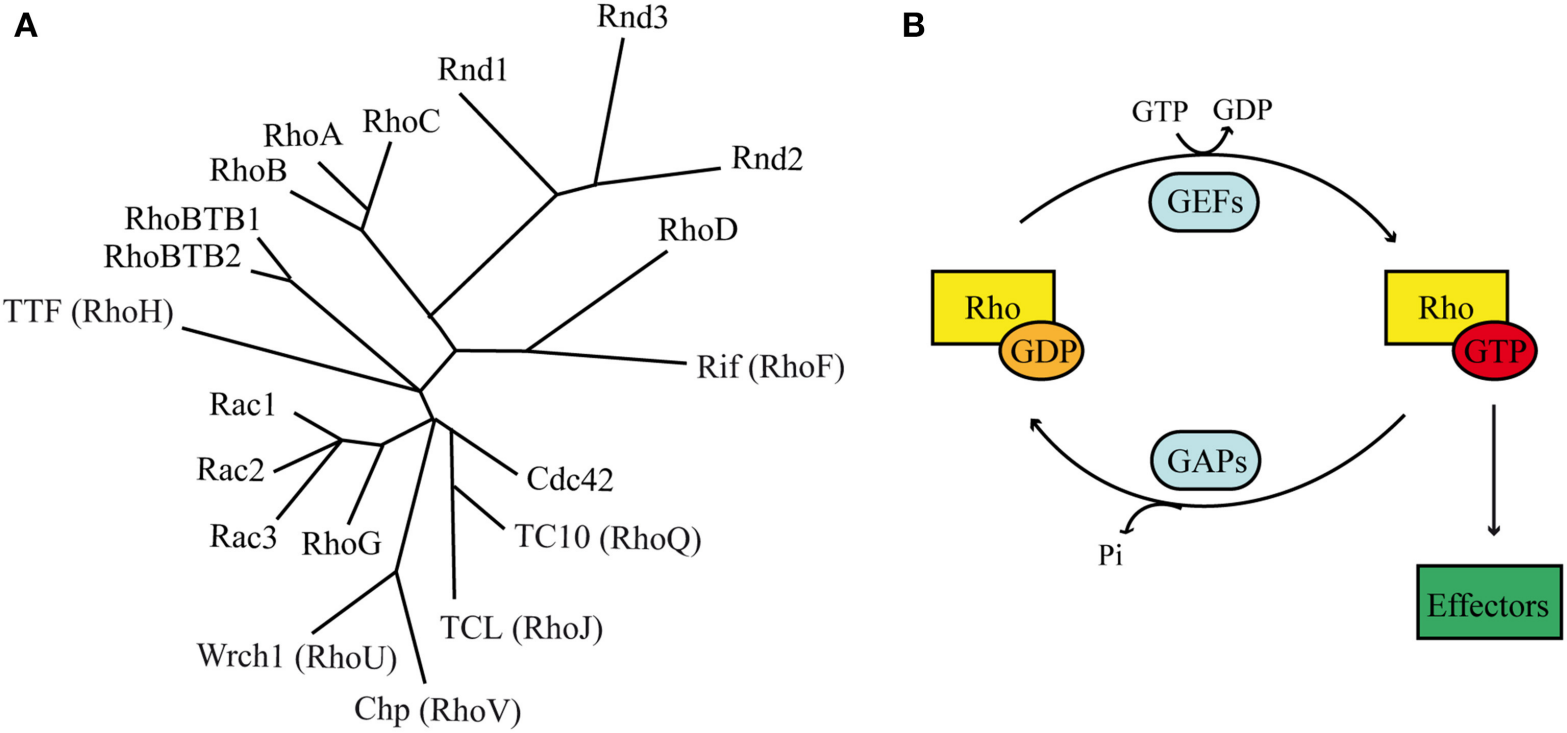
**Rho GTPases. (A)** Phylogenetic tree based on alignment of the aminoacid sequences of the 20 Rho GTPases. Rnd proteins form a distinct branch, which is closely related to Rho members. **(B)** Classical Rho GTPases cycle between an inactive GDP-bound state and an active GTP-bound state. In their active conformation they transduce the signal to intracellular effectors. Two classes of molecules promote the regulatory cycle: GEFs stimulate the exchange of GDP with GTP, whereas GAPs stimulates the GTP hydrolysis.

Rho (Ras homologous) GTPases belong to the large superfamily of small GTP binding proteins, whose founding member is Ras (Jaffe and Hall, 2005; Heasman and Ridley, 2008). Ras superfamily contains more than 150 members, which are grouped into 5 categories according to their major functions: Ras, Rho, Rab, Arf, and Ran (Table 1). Mammalian Rho GTPases comprise a family of 20 molecules that regulate actin and microtubule components of the cytoskeleton (Figure 2A). By controlling cytoskeletal dynamics, Rho GTPases affect many cellular processes, including cell polarity, cell shape and migration (Hall and Nobes, 2000; Ridley, 2001). The most extensively studied members of the Rho family are RhoA (Ras homologous member A), Rac1 (ras related C3 botulinum toxin substrate 1) and Cdc42 (cell division cycle 42). Rac1 and Cdc42 promote the formation of cellular protrusions, such as lamellipodia or filopodia, respectively. RhoA instead is involved in acto-myosin contraction and stress fiber formation (Ridley, 2001). The overexpression of constitutively active or dominant negative forms of Rho proteins in the embryonic cortex, together with more recent analysis of conditional knockout mice have revealed a crucial role for Rac1 and Cdc42 during INM (Cappello et al., 2006; Minobe et al., 2009) and for RhoA, Rac1, and Cdc42 during radial migration in the cortex (Kawauchi et al., 2003; Konno et al., 2005; Cappello et al., 2012). (For recent reviews see (Govek et al., 2011; Shah and Puschel, 2014).

**Table 1:** **Members of the Ras superfamily and their major functions**.

**Family**	**Members**	**Function**
Ras	Ha-Ras, K-Ras, N-Ras, R-Ras, M-Ras, RalA, RalB, Rap1A, Rap1B, Rap2A, TC21, Rit, Rin, Rad, Kir/Gem, Rheb, KB-Ras1, KB-Ras2	Control of cell proliferation
Rho	RhoA, RhoB, RhoC, RhoD, Rif (RhoF), Rnd1 (Rho6), Rnd2 (Rho7, RhoN), Rnd3 (Rho8, RhoE), TTF (RhoH), Rac1, Rac2, Rac3, RhoG, Cdc42, TC10 (RhoQ), TCL (RhoJ), Wrch1 (RhoV), Chp/Wrch2 (RhoU), RhoBTB1, RhoBTB2	Control of cell cytoskeleton
Rab	Rab proteins from Rab1 to Rab33	Control of vesicle trafficking
Arf	Arf1, Arf2, Arf3, Arf4, Arf5, Arf6, Sar1a, Sar1b, Arl1, Arl2, Arl3, Arl4, Arl5, Arl6, Arl7, Ard1	Control of vesicle formation
Ran	Ran	Control of nuclear transport

Most Rho GTPases act as molecular switches by cycling between an inactive GDP-bound state and an active GTP-bound form (Figure 2B). When bound to GTP, Rho GTPases exhibit the correct structural conformation to interact with effectors and initiate downstream signaling (Raftopoulou and Hall, 2004). The GDP/GTP cycle is promoted by the activity of two classes of molecules, guanine nucleotide exchanging factors (GEFs) and GTPase activating proteins (GAPs). GEFs facilitate the exchange of GDP with GTP, resulting in protein activation. GAPs instead stimulate the intrinsic enzymatic activity of the GTPases, which promotes hydrolysis of GTP into GDP. GAP activity therefore ends the cycle and returns the GTPases in their inactive state (Bos et al., 2007). In addition, Rho GTPases can bind to proteins known as guanine-nucleotide dissociation inhibitors (GDIs). RhoGDIs sequester RhoGTPase in their inactive state and protect them from degradation (Dermardirossian and Bokoch, 2005; Boulter et al., 2010).

The GDP/GTP cycle and the regulation by GDI are common properties among Rho GTPases. However, the “atypical” Rho GTPases rarely follow this rule (Aspenstrom et al., 2007). Among them, the Rnd subfamily represents a distinct branch of the Rho family of small GTPases and consists of three different members: Rnd1/Rho6, Rnd2/Rho7, and Rnd3/Rho8/RhoE (Chardin, 2006; Riou et al., 2010) (Figure 2A). Interestingly, the Rnd subfamily is present only in vertebrates and not in other organisms such as worms or flies, suggesting that it might play important roles in biological processes that are specific to vertebrate organisms (Philips et al., 2003; Boureux et al., 2007). Rnd proteins have a core GTP-binding domain structurally similar to the other Rho proteins. However, they possess different biochemical properties due to amino acid substitutions at residues that are crucial for GTPase activity. In fact, in contrast to classical GTPases, Rnd proteins do not show any intrinsic or stimulated GTPase activity. In addition to their inability to hydrolize GTP into GDP, Rnd proteins exhibit a 100-times higher affinity for GTP than for GDP. Altogether, these properties suggest that Rnd are constitutively bound to GTP, therefore constitutively active (Foster et al., 1996; Nobes et al., 1998). Nobes et al. (1998) were the first to identify the three Rnd isoforms and shed light onto their function. By overexpressing Rnd1 and Rnd3 in cultured fibroblasts, the authors observed cell retraction from the substrate and cell rounding, hence their collective name round (Rnd). This phenotype is the result of Rnd1 and Rnd3 inhibitory functions on RhoA-mediated stress fiber formation and adhesion contact assembly. In contrast to Rnd1 and Rnd3, the expression of Rnd2 in fibroblasts does not modulate cytoskeletal reorganization, suggesting that Rnd2 acts via different and partially unknown mechanisms in these cells (Nobes et al., 1998). Recent evidences demonstrate that Rnd3 also plays a role in the control of cell proliferation via mechanisms that are independent from cytoskeletal remodeling (Villalonga et al., 2004; Poch et al., 2007; Pacary et al., 2013), indicating that Rnd proteins might have more pleiotropic functions that previously expected. Among the three members of the Rnd subfamily, only *Rnd2* and *Rnd3* show strong expression in the developing cerebral cortex. *Rnd2* is found in the preplate cells at early stages of cortical development and it is expressed in the SVZ/IZ at later stages (Heng et al., 2008). In contrast to *Rnd2, Rnd3* expression is widespread throughout the entire thickness of the cortical wall at early stages and it is later restricted to the VZ/SVZ, as well as to the CP (Pacary et al., 2011). The distribution of *Rnd2* and *Rnd3* transcripts in partially exclusive cortical domains suggests that they might play individual and non-redundant functions in distinct phases of cortical development and neuronal migration.

## Rnd functions in cortical neuron migration

### Rnd3 role in interkinetic nuclear migration

The role of Rnd3 in INM has been recently studied *in vivo*, by *in utero* electroporation of the embryonic cortex with shRNA that specifically silences *Rnd3* expression (Pacary et al., 2013). The process of INM can be visualized and quantified after injection of 2-bromo-deoxyuridine (BrdU), which marks cells in S phase, followed by analysis of the position of the BrdU positive nuclei over time (Schenk et al., 2009). The nature of INM implies that cells that are in S phase at the time of BrdU injection are positioned in the most basal region of the VZ. BrdU labeled cells can be then followed when they subsequently undergo basal-to-apical nuclear migration to reach the apical surface, just before entering mitosis. Analysis performed 30 min after BrdU injection revealed that, in *Rnd3*-silenced cortices, a reduced fraction of BrdU^+^ nuclei reach the apical side in comparison to control treated cortices, indicating delayed nuclear migration when *Rnd3* expression is decreased in progenitor cells (Pacary et al., 2013). Three hours after injection, control BrdU labeled cells have undergone cell division at the ventricular surface and the nucleus of the radial glia daughter cell begins to move again toward the basal side. In contrast, the delayed nuclei in *Rnd3* knock down cortices have just reached the apical side and start to divide, leading to an accumulation of cells at the ventricular surface. In addition, Rnd3-silenced VZ progenitors exhibit less elongated nuclei compared to control cells (Pacary et al., 2013), which is characteristic of INM impairment (Sauer, 1935; Ge et al., 2010) Altogether these data show that Rnd3 is required during INM at least for the basal to apical movement. Importantly, the duration of the different phases of the cell cycle is unaffected by *Rnd3* silencing, indicating that the regulation of INM by Rnd3 is direct and not secondary to modification of cell-cycle progression in neural progenitors.

### Rnd functions in radial migration of projection neurons

The role of Rnd proteins in cortical neuron migration has been thoroughly investigated only in the last few years (Nakamura et al., 2006; Heng et al., 2008; Pacary et al., 2011; Azzarelli et al., 2014). As mentioned before, *Rnd2* and *Rnd3* are expressed in different cortical domains during embryonic development suggesting that they might control different phases of the migratory process. Accordingly, the *in vivo* knock down of Rnd2 and Rnd3 in the embryonic cortex produces migratory defects that are characterized by distinct morphological abnormalities.

**Figure 3:**
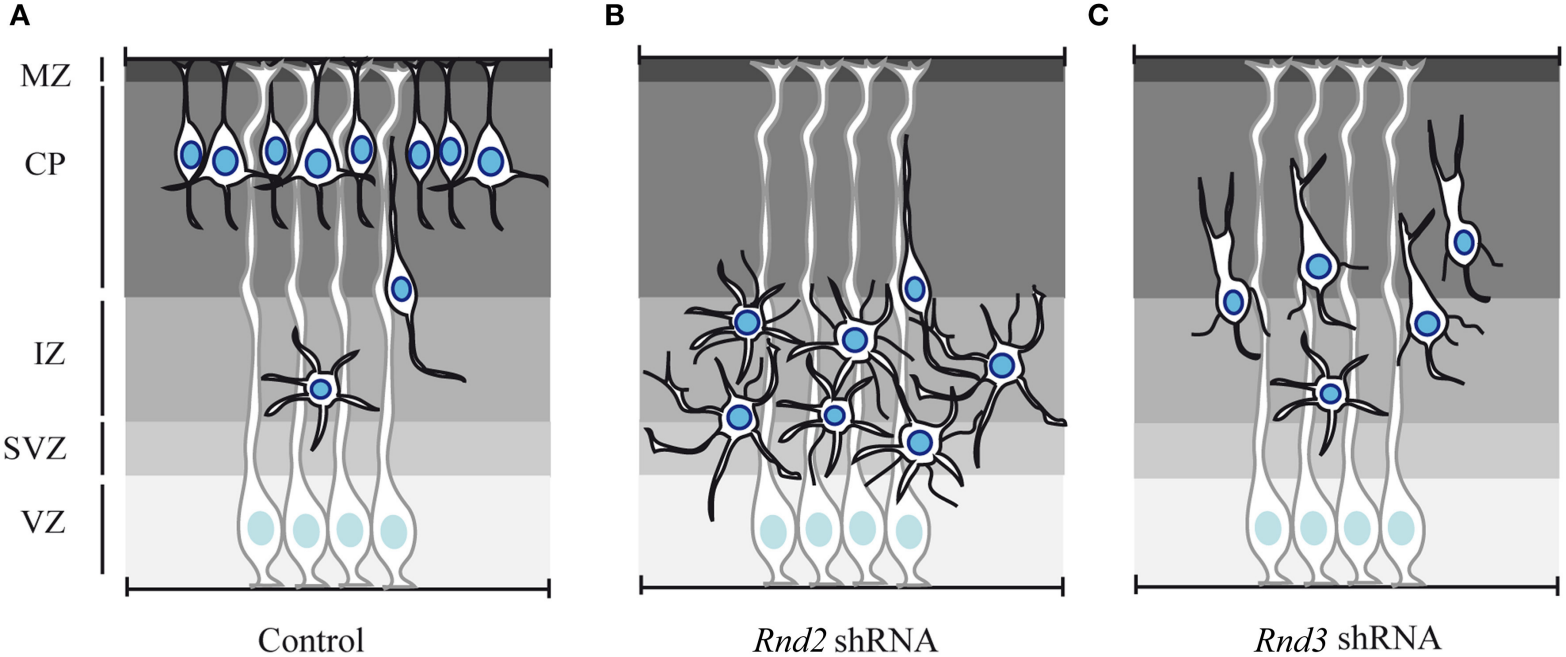
**Effect of *Rnd2* and *Rnd3* loss of function on cortical neuron migration. (A)** Schematic representation of cortical radial migration in control condition. Newborn projection neurons undergo sequential steps of radial migration, which are characterized by distinct morphologies. At mid-end corticogenesis most of the neurons have reached the CP. Only few cells are still migrating and they exhibit multipolar morphology in the IZ and bipolar shape in the CP. **(B)** shRNA-mediated loss of function of *Rnd2* expression in the embryonic cortex produces an accumulation in the IZ of multipolar cells, which exhibit more and longer neuronal processes. **(C)** *Rnd3* knock down in the embryonic cortex interferes with the locomotion phase of migration in the CP. *Rnd3*-silenced neurons exhibit abnormal morphologies characterized by excessively enlarged and branched leading processes and by thin processes protruding from the cell body. MZ, marginal zone; CP, cortical plate; IZ, intermediate zone; SVZ, subventricular zone; VZ, ventricular zone.

As neurons progress from the VZ/SVZ to the CP, they transiently acquire a multipolar morphology in the IZ. *In vivo* knock down of *Rnd2*, but not of *Rnd3* expression, increases the fraction of neurons with a multipolar shape. This phenotype eventually leads to the accumulation of cells in the IZ of *Rnd2*-knocked down cortices and a concomitant reduction of neurons reaching the CP, in comparison to control cortices (Figures 3A,B) (Heng et al., 2008). Rnd2 thus regulates multipolar to bipolar transition in the IZ.

*Rnd3*-silenced neurons instead exhibit abnormal morphology during neuronal migration in the CP, i.e., during glia-guided locomotion. During this phase, migrating neurons in control condition exhibit a bipolar morphology with a leading process toward the CP and a trailing process in the direction of the IZ. In contrast, Rnd3-knocked down neurons display a grossly enlarged leading process and several thin processes protrude from the cell body (Figure 3C). Locomotion in the CP largely relies on the coordinated movement of nucleus and centrosome in the direction of migration. Neurons undergo cycles of extension of the leading process and forward movement of the nucleus toward the centrosome, which is located in a cytoplasmic dilation that forms in the proximal region of the leading process. When *Rnd3* expression is reduced by shRNA electroporation, the distance between the nucleus and the centrosome in bipolar neurons is increased. A possible role for Rnd3 in the regulation of nucleus-centrosome coupling during locomotion has been further supported by *ex vivo* time-lapse imaging, which clearly shows that the motility behavior of *Rnd3*-depleted neurons is not coordinated (Pacary et al., 2013).

Consistent with the aberrant migration in the CP, *Rnd3*-silenced neurons also exhibit a branched leading process, which have been previously associated with loss of adhesion between the leading process and the radial glia fibers (Gupta et al., 2003; Elias et al., 2007). Whether *Rnd3*-silenced neurons are detached from the radial glia scaffold and whether loss of adhesion is a primary effect or secondary to defective locomotion would be interesting issues to address in the future.

## Regulation of Rnd proteins

Rnd proteins are always present in the cell in their active conformation, capable to bind effectors. Since their activity is not affected by the GDP/GTP exchange or by interaction with RhoGDIs, other mechanisms must account for the regulation of their activity (Riou et al., 2010). Transcriptional regulation, subcellular localization and post-translational modifications have been shown to play crucial roles in the control of Rnd protein expression and function.

During the development of the cerebral cortex, *Rnd2* and *Rnd3* are under the transcriptional control of proneural factors that up-regulate their expression to control specific phases of neuronal migration. The proneural factors Neurogenin2 (Neurog2) and Ascl1 directly bind to E-box DNA sequences within enhancers located 3′ to the *Rnd2* and *Rnd3* gene, respectively (Heng et al., 2008; Pacary et al., 2011). Moreover, *Rnd2* expression in the developing brain is transcriptionally regulated by other factors, such as RP58, Scratch2, and COUP-TFI, which act as repressors (Alfano et al., 2011; Heng et al., 2013; Ohtaka-Maruyama et al., 2013; Paul et al., 2014). In particular, RP58 and Scratch2 regulate the 3′ enhancer previously identified as Neurog2 target, suggesting that the two repressors might compete with the proneural bHLH activator on *Rnd2* enhancer to fine-tune the levels of *Rnd2* in the cortex (Heng et al., 2013). Several other studies in different cell types have identified various stimuli that regulate Rnd2 and Rnd3 expression (Table 2).

**Table 2:** **Mechanisms regulating Rnd expression**.

**Rnd**	**Stimuli or TFs that control expression**	**Cell type**	**Function**	**References**
Rnd2	Neurog2	Cortical neurons	Migration	1
Rnd2	RP58*	Cortical neurons	Migration	2
Rnd2	COUPTFI*	Cortical neurons	Migration and differentiation	3
Rnd2	Scratch2*	Cortical neurons	Migration	4
Rnd3	Ascl1	Cortical neurons	Migration	5
Rnd3	PDGF	Fibroblast	Formation of stress fibers	6
Rnd3	HGF	MDCK	Motility	7, 8
Rnd3	Raf-MEK-BRF	MDCK	Transformation	9
		Melanoma cells	Invasiveness	10, 11
Rnd3	p53—chemoterapeutic agent or irradiation	Cancer cell line, keratinocytes	Pro-survival	12, 13
Rnd3	mTOR	Subependymal giant cell astrocytoma	Potential contribution to tumorigenesis	14
Rnd3	NF-kB	Prostate cancer	Potential contribution to tumorigenesis	15
Rnd2/3	MDMA and cocaine	Neurons in different brain regions	Potential contribution to dendritic branching and neurite outgrowth	16
Rnd3	mir200c mir200b*	Breast cancer cell	Invasive behavior	17, 18
Rnd1-2-3	Estradiol	Smooth muscle cells—myometrium	Decreased contraction	19
Rnd3	Estradiol*	Prostatic stromal cells	Unknown	20
Rnd3	MIC-1/GDF15	Prostate cancer cells	Decreased adhesion	21
Rnd3	CREB	Hippocampal neurons	BDNF-mediated synaptogenesis	22
Rnd3	HIF1a	Gastric cancer cells	Epithelial to mesenchymal transition and invasion	23
Rnd3	FOXD3*	Melanoma cells	Migration and invasion	24

1 (Heng et al., 2008), 2 (Heng et al., 2013; Ohtaka-Maruyama et al., 2013), 3 (Alfano et al., 2011), 4 (Paul et al., 2014), 5 (Pacary et al., 2011), 6 (Riento et al., 2003), 7 (Guasch et al., 1998), 8 (Tanimura et al., 2002), 9 (Hansen et al., 2000), 10 (Klein et al., 2008), 11 (Klein and Aplin, 2009), 12 (Ongusaha et al., 2006), 13 (Boswell et al., 2007), 14 (Tyburczy et al., 2010), 15 (Nadiminty et al., 2010), 16 (Marie-Claire et al., 2007), 17 (Hurteau et al., 2006), 18 (Xia et al., 2010), 19 (Shimomura et al., 2009), 20 (Bektic et al., 2004), 21 (Liu et al., 2003), 22 (Lesiak et al., 2013), 23 (Zhou et al., 2011), 24 (Katiyar and Aplin, 2011). ^*^Denotes factors that decrease Rnd expression.

Rnd proteins also undergo post-translational modifications that influence their subcellular localization and stability. Most Rho GTPases are modified at their C-terminus by addition of lipid moieties that promote their interaction with membranes (Seabra, 1998). Whereas most Rho-family proteins are geranylgeranylated, Rnd proteins are farnesylated, which consists in the addition of a 15-carbon farnesyl group on their C-terminal CAAX motif (where C represents cysteine, A is an aliphatic amino acid and X is any amino acid). This motif is important not only for membrane localization but also for Rnd activity. Indeed, mutation in the CAAX motif of Rnd3 (Rnd3^C241S^) abolish its plasma membrane association and impairs its ability to rescue the migratory activity of *Rnd3*-silenced neurons, thus demonstrating that membrane association is required for Rnd3 activity in migrating neurons (Pacary et al., 2011). In addition to this motif, sequence elements positioned immediately upstream of the CAAX domain are important for membrane insertion (Roberts et al., 2008). Rnd2 and Rnd3 have similar CAAX motif, but distinct upstream sequences that are responsible for the different subcellular localization of Rnd2 and Rnd3. In fact, Rnd3 is preferentially associated to the plasma membrane, whereas Rnd2 is cytoplasmic or associated to endomembranes (Roberts et al., 2008). It has been recently shown that the replacement of the C-terminal domain of Rnd2, containing the CAAX motif and the upstream sequence, with that of Rnd3 is sufficient to recruit Rnd2 at the plasma membrane (Pacary et al., 2011). More importantly, although Rnd2 and Rnd3 cannot substitute for one another during cortical neuron migration, the chimeric form of Rnd2 that exhibits a plasma membrane localization similar to Rnd3 can compensate for the loss of *Rnd3* (Pacary et al., 2011). Hence, when targeted to the correct subcellular region, Rnd2 can replace Rnd3 function in migrating neurons. Recent evidence also supports a role for the N-terminal region of Rnds in the control of subcellular localization. There is indeed a specific sequence at the N-terminal of Rnd1 and Rnd3, but not of Rnd2, that promotes their targeting to specialized membrane regions, the lipid rafts (Oinuma et al., 2012).

Another important post-translational modification that regulates Rnd protein activity is phosphorylation. Seven phosphorylation sites have been identified in Rnd3 (5 at the C-terminal end and 2 at the N-terminal end) and they have been shown to influence Rnd3 localization at the plasma membrane. Rnd3 can be phosphorylated by ROCKI or PKCα on multiple sites (Riento et al., 2005; Komander et al., 2008; Madigan et al., 2009) and upon phosphorylation, Rnd3 affinity for plasma membrane is reduced and the fraction of cytosolic Rnd3 increases. Interestingly, a non-phosphorylatable mutant form of Rnd3 (Rnd3^All A^) that is preferentially associated to the plasma membrane is more efficient than wild-type Rnd3 in rescuing the cortical migration defects induced by *Rnd3* silencing (Madigan et al., 2009; Pacary et al., 2011). This result further demonstrates that the membrane association of Rnd3 regulates its activity in migrating neurons and determines the efficiency with which neurons migrate in the embryonic cortex.

Classical Rho proteins are generally solubilized from the plasma membrane and sequestered inactive in the cytosol, by interaction with RhoGDIs that mask the un-soluble hydrophobic group. Since Rnd proteins do not interact with RhoGDIs, an alternative mechanism has recently been proposed to explain how phosphorylated Rnd proteins become internalized and solubilized in the cytosol (Riou et al., 2013). Anne Ridley and colleagues have demonstrated that upon phosphorylation, the C-terminal region of the three Rnd interacts with the regulatory molecules 14-3-3. This interaction masks the lipid moiety of the Rnd protein and permit translocation from the plasma membrane to the cytosol. Whether the localization of Rnd2 and Rnd3 and thus their activity is controlled by this mechanism in cortical neurons is not known.

Lastly the levels of Rnd proteins in a cell can be controlled by their effectors through protein stabilization. It has been shown that the binding of Rnd3 to its effectors stabilizes Rnd3 proteins, suggesting that a positive feedback from effectors may contribute to extend the half-life of Rnd (Goh and Manser, 2012). This mechanism of regulation remains to be studied in migrating cortical neurons.

Altogether, the variety of factors that controls Rnd protein expression and localization reveal that Rnd activity is regulated by complex mechanisms, which substitute for the lack of the classical GDP/GTP molecular switch and GDI internalization.

## Molecular mechanisms mediating Rnd activity in migrating neurons

### Regulation of RhoA signaling and cytoskeleton remodeling

Experiments performed in non-neuronal cell types revealed that a mechanism commonly used by Rnd proteins to control cytoskeletal dynamics is the inhibition of RhoA signaling (Nobes et al., 1998; Wennerberg et al., 2003; Riou et al., 2010). Similarly, FRET analysis demonstrated that RhoA activity is increased in migrating neurons after *Rnd2* or *Rnd3* knockdown (Pacary et al., 2011). More importantly, in this study, coelectroporation of *Rnd3* shRNA together with a *RhoA* shRNA fully rescue the radial migration of *Rnd3*-silenced neurons, thus demonstrating that Rnd3 regulate radial migration in the cortex mostly by inhibiting RhoA activity. The same kind of experiment performed with *Rnd2* shRNA indicates that this RhoGTPase, in contrast to Rnd3, acts only partially through suppression of RhoA activity in migrating neurons.

In fibroblasts and epithelial cells, Rnd-mediated inhibition of RhoA activity induces cell rounding via disassembly of stress fibers, which are composed of bundles of actin filaments. Although neurons do not possess stress fibers, Rnd proteins have been shown to also control the dynamics of filamentous actin (F-actin) in migrating neurons (Pacary et al., 2011). In cultured primary cortical neurons, both *Rnd2* and *Rnd3* knock down produce an accumulation of F-actin in neuronal processes as well as in the cell body in the case of Rnd2. A common pathway that controls F-actin polymerization downstream of RhoA signaling is the ROCK (Rho Kinase)- LIMK (Lim Kinase)—cofilin pathway (Maekawa et al., 1999; Sumi et al., 1999; Peris et al., 2012). The activation of RhoA ultimately phosphorylates and inactivates cofilin, which is an actin-disassembling factor, thus resulting in local increase of F-actin. The co-electroporation in the embryonic cortex of a non-phosphorylatable form of cofilin (cofilin^S3A^), which constitutively depolymerizes actin, together with *Rnd3* shRNA completely rescues the migration defects induced by *Rnd3* silencing. This suggests that when *Rnd3* is silenced, the RhoA-cofilin-mediated excessive polymerization of actin molecules hampers the motility behavior of migrating neurons. Interestingly, co-electroporation of the cofilin mutant with *Rnd3* shRNA also rescues the defects that *Rnd3* silencing produces during INM in VZ progenitor cells (Pacary et al., 2013), suggesting that similar basic molecular mechanisms may control nuclear translocation during INM and glia-guided locomotion.

In contrast, the migratory defects induced by *Rnd2* knockdown are not rescued by the mutated form of cofilin, indicating that Rnd2 promotes migration independently of its effect on the actin cytoskeleton. It is possible that accumulation of F-actin and aberrant cytoskeletal organization upon *Rnd2* knock down might be secondary to other events that impede migration. Rnd2 has been shown to be expressed in endosomes and to interact with molecules involved in the formation and trafficking of endocytic vesicles (Fujita et al., 2002; Tanaka et al., 2002; Wakita et al., 2011), raising the possibility that Rnd2 pro-migratory activity may involve the regulation of endocytosis. Further studies will be required to test this hypothesis.

Consistent with their different activities, Rnd2 and Rnd3 cannot replace one another, even if they both inhibit RhoA signaling. This apparent paradox can be explained by the fact that Rnd2 and Rnd3 interfere with RhoA activity in different subcellular compartments (Pacary et al., 2011). Indeed, Rnd3 preferentially localizes at the plasma membrane and inactivates RhoA in this compartment, whereas Rnd2 is expressed only in endosomes and cytosol, confining RhoA regulation to these internal structures (Pacary et al., 2011). In accordance with these data, Rnd2 can replace Rnd3 function in migrating neurons if it is targeted to the plasma membrane by replacement of its C-terminal region with the one of Rnd3, as already mentioned. Importantly, the reduction of RhoA activity in endosomes has been shown to be essential for clathrin mediated endocytosis (Lamaze et al., 1996; Qualmann and Mellor, 2003; Ridley, 2006), further reinforcing the hypothesis that Rnd2 might control cortical neuron migration by regulating the trafficking of receptors or adhesion molecules which are essential for this process. However, it is worth noting that Rnd2 inhibition of RhoA signaling cannot fully explain Rnd2 pro-migratory activity and therefore Rnd2 might act in the cortex also via a different and RhoA-independent mechanism (Pacary et al., 2011).

### Mechanisms of RhoA regulation by Rnd proteins

**Figure 4:**
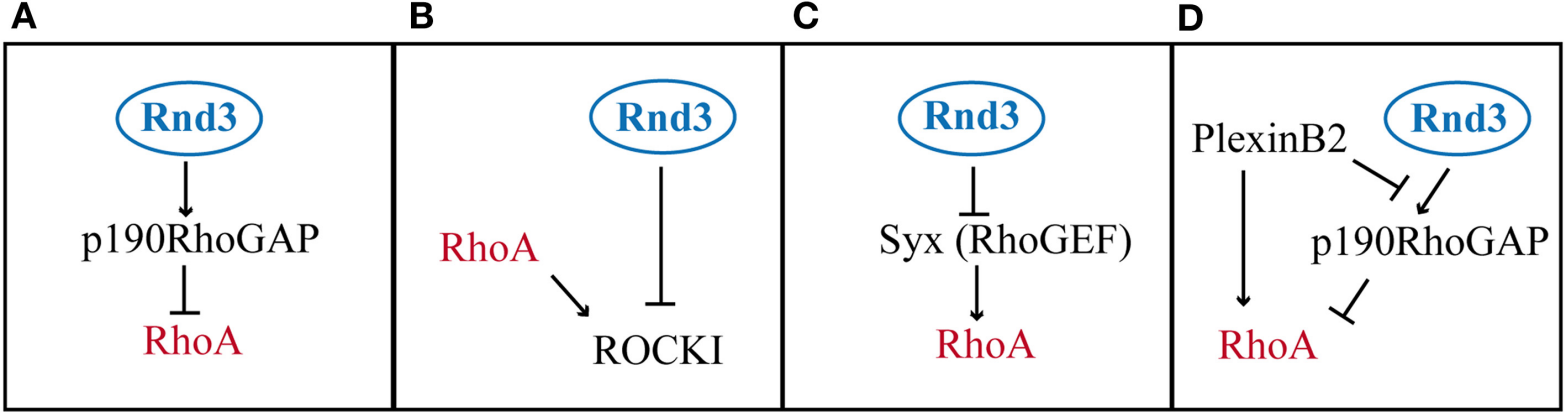
**Modes of RhoA activity regulation by Rnd3. (A)** Rnd3 interacts with p190RhoGAP and promotes its activity of RhoA inactivation. **(B)** Rnd3 indirectly inhibits RhoA signaling, by blocking the RhoA downstream effector ROCKI. **(C)** Rnd3 inhibits a RhoA activator like Syx. **(D)** PlexinB2 interaction with Rnd3 disrupts Rnd3-p190RhoGAP binding, which lifts RhoA inhibition. In addition PlexinB2 directly activates RhoA via recruitment of RhoGEFs (not shown).

The molecular bases for Rnd-mediated RhoA inhibition are not yet completely understood, but many evidences suggest the existence of various mechanisms (Figure 4). Rnd3, for example, has been shown to antagonize RhoA signaling via three different pathways: (1) by promoting the activity of RhoA GAPs, which promote the hydrolysis of the GTP into GDP, such as p190RhoGAP (Wennerberg et al., 2003) (Figure 4A, Table 3), (2) by blocking the activity of RhoA effectors, such as the Rho kinase ROCKI (Riento et al., 2003) (Figure 4B, Table 3); (3) by inhibiting Rho GEFs, which exchange GDP with GTP on RhoA, such as Syx (Goh and Manser, 2010) (Figure 4C, Table 3).

**Table 3:** **Rnd interacting partners and their functions**.

**Rnd partner**	**Rnd**	**Function**	**References**
p190RhoGAP	Rnd1	Down-regulation of RhoA in stress fiber collapse and during cortical neuron migration	1, 2, 3, 4
	Rnd2		
	Rnd3		
ROCKI	Rnd3	Inhibition of ROCKI signaling in stress fiber disassembly; control of Rnd localization and stability through phosphorylation	5, 6
Syx	Rnd3	Down-regulation of RhoA in Zebrafish gastrulation	7
MgcRacGAP	Rnd2	Regulation of RhoGTPase flux during cytokinesis; control of male germ cell development	8, 9
PlexinB2	Rnd3	Regulation of neuronal migration by fine-tuning RhoA signaling	4
PlexinB1	Rnd1	Activation of RhoA and down-regulation of R-Ras in growth cone collapse	10, 11
PlexinD1	Rnd2	Down-regulation of R-Ras in axon outgrowth inhibition	12
PlexinA1	Rnd1	Activation of Rac1 and down-regulation of R-Ras in axonal repulsion	13, 14, 15
Rapostilin	Rnd2	Regulation of endocystosis and membrane invagination in neurite branching and spine formation	16, 17
Vps4A	Rnd2	Regulation of endosomal trafficking	18
FLRT3	Rnd1	Control of cadherin-mediated adhesion during Xenopus gastrulation	19, 20
Pragmin	Rnd2	Activation of RhoA in neurite outgrowth inhibition	21
SCG10	Rnd1	Control of microtubule stability in axon formation	22
Socius	Rnd1	Loss of stress fibers	23
FSR2a/b	Rnd1	Control of neurite extension downstream of FGF signaling	24
Grb7	Rnd1	Possible role in migration/invasion	25
STI1	Rnd1	Control of cytoskeletal collapse	26

*1 (Foster et al., 1996), 2 (Wennerberg et al., 2003), 3 (Pacary et al., 2011), 4 (Azzarelli et al., 2014) 5 (Riento et al., 2003), 6 (Riento et al., 2005), 7 (Goh and Manser, 2010), 8 (Naud et al., 2003), 9 (Miller and Bement, 2009), 10 (Oinuma et al., 2003), 11 (Oinuma et al., 2004), 12 (Uesugi et al., 2009), 13 (Rohm et al., 2000), 14 (Toyofuku et al., 2005), 15 (Zanata et al., 2002), 16 (Fujita et al., 2002), 17 (Wakita et al., 2011), 18 (Tanaka et al., 2002), 19 (Karaulanov et al., 2009), 20 (Ogata et al., 2007), 21(Tanaka et al., 2006), 22 (Li et al., 2009), 23 (Katoh et al., 2002), 24 (Harada et al., 2005), 25 (Vayssiere et al., 2000), 26 (De Souza et al., 2014)*.

The first mechanism, which involves the stimulation of p190RhoGAP by Rnd3, seems to be an important pathway of RhoA inhibition downstream of Rnd proteins, since Rnd1 and Rnd2 have also been shown to interact with p190RhoGAP (Wennerberg et al., 2003; Pacary et al., 2011). However, a mutant form of Rnd2 (Rnd2^T39V^), which cannot bind to p190RhoGAP, is as active as wild type Rnd2 in rescuing the neuronal migration defects induced by *Rnd2* silencing in the cortex. Therefore, even if Rnd2 can interact with p190RhoGAP, this interaction does not mediate Rnd2 function in this context. It is possible that Rnd2 works via interaction with a different RhoGAP. One candidate is MgcRacGAP, which has been found associated to Rnd2 in male germ cells (Naud et al., 2003) and which is expressed in the developing cerebral cortex at the time of radial migration (Arar et al., 1999) (Table 3). MgcRacGAP (or RacGAP1) primary targets are Rac1 and Cdc42, but upon phosphorylation, MgcRacGAP turns its activity toward RhoA (Toure et al., 2008). Rather than promoting mere inhibition of RhoA activity, MgcRacGAP has been shown to control a RhoA GTPase flux at the site of furrow formation during cytokinesis (Miller and Bement, 2009). In the future, it would be interesting to understand whether MgcRacGAP mediates Rnd2 function in the endosomal compartments and to study whether also Rnd3 uses this different RhoGAP during early steps of corticogenesis, when Rnd3 is known to control INM, cleavage plane orientation, VZ integrity and SVZ progenitor proliferation (Pacary et al., 2013).

In contrast to Rnd2, Rnd3 requires the interaction with p190RhoGAP for its pro-migratory activity in the cortex. Indeed, an Rnd3 mutant form (Rnd3^T55V^) that cannot bind to p190RhoGAP in rescue experiments failed to replace Rnd3 function in migrating neurons (Pacary et al., 2011). However, it has been recently shown that this Rnd3 mutant carries a mutation in the effector binding domain (Wennerberg et al., 2003), which not only prevents Rnd3 from binding to p190RhoGAP but also disrupts the ability of Rnd3 to bind to other candidate effectors, including a member of the Plexin family of axon guidance receptors, PlexinB2 (Azzarelli et al., 2014) (Table 3). Therefore, it is possible that Rnd3 activity in the cortex may also require the interaction with effectors other than p190RhoGAP. The binding of Rnd3 to the RhoA effector ROCKI is however not affected by the T55V mutation in the effector domain. Instead, ROCKI binds Rnd3 in a different position and ROCKI-Rnd3 interaction can be selectively disrupted by mutation of two sites present in the C-terminal region of Rnd3 protein (Rnd3^T173A+V192A^) (Wennerberg et al., 2003). Selective disruption of Rnd3-ROCKI interaction does not interfere with Rnd3 function in migrating neurons, which indicates that blocking ROCKI does not account for Rnd3 inhibition of RhoA activity in this context. Finally, whether Rnd2 or Rnd3 also modulate RhoA activity in migrating neurons via Syx or other RhoGEFs remains unexplored.

### A role for plexins

Over the past few years, Rnd proteins have been shown to constitute important functional components of the plexin-semaphorin signaling pathways (Chardin, 2006; Puschel, 2007). Plexins belong to a large family of transmembrane receptors, which are activated by their physiological ligands, the semaphorins. In vertebrates, there are 9 plexin members, which can be divided into four classes, termed plexinA (A1–A4), B (B1–B3), C1 and D1 and 7 classes of secreted and membrane-bound semaphorins (Jackson and Eickholt, 2009). Although plexin-semaphorin signaling has been historically associated with regulation of axonal navigation, novel roles during brain developmental and neuronal migration have started to be characterized (Luo et al., 1993; Comeau et al., 1998; Kruger et al., 2005; Pasterkamp, 2012).

Plexins contain a binding site for Rho GTPases in the middle of their intracellular domain through which they recruit several Rho GTPases, including Rnd proteins. Several evidences indicate that preferential interactions occur between certain members of the Plexin and the Rnd families (Table 3). For example, PlexinB1 binds to Rnd1 and Rnd2, but not to Rnd3, which instead selectively interacts with PlexinB2 (Oinuma et al., 2003; Azzarelli et al., 2014); also, PlexinD1 has been found associated only with Rnd2, but not with Rnd1 or Rnd3 (Uesugi et al., 2009) and PlexinA1 interacts with Rnd1, but not with Rnd2 (Zanata et al., 2002). The functional relevance of the exclusive Plexin-Rnd interactions is not clear, but it is likely that the recruitment of specific Rnds may be important to differentially modulate plexin signaling.

Rnd1 binding to PlexinB1 has been shown to open the conformation of the receptor and to allow the transmission of the downstream signaling. This synergistic interaction is essential to drive cell contraction in COS cells and to induce growth cone collapse during axon guidance (Chardin, 2006). In migrating cortical neurons, the interaction between Rnd3 and PlexinB2 is crucial to fine-tune the levels of active RhoA. PlexinB2 recruitment of Rnd3 to its intracellular domain disrupts the interaction between Rnd3 and p190RhoGAP in a competitive manner. In this way, PlexinB2 blocks Rnd3-mediated RhoA inhibition and it has been proposed that this step is required for full RhoA activation in specific cellular compartments (Azzarelli et al., 2014) (Figure 4D). Therefore, through competitive Rnd3 binding, p190RhoGAP and PlexinB2 fine-tune the level of RhoA activity appropriate for cortical neuron migration.

Rnd2 has also been found associated with Plexin members like PlexinB1 and PlexinD1. However, in contrast to Rnd3, which co-localizes with PlexinB2 at the plasma membrane in primary cortical neurons, Rnd2 is not found at the plasma membrane (Pacary et al., 2011), therefore making it unlikely that Rnd2 plays a part in plexin downstream signaling that is activated in this subcellular compartment. Instead, Rnd2 is expressed in early endosomes (Pacary et al., 2011), where it interacts with Fbp17/Rapostlin and Vps4, two molecules involved in the formation and the trafficking of endocytic vesicles (Table 3) (Fujita et al., 2002; Tanaka et al., 2006). Therefore, Rnd2 potential interaction with plexins may be an important strategy to control the surface expression of these receptors through the stimulation of their endocytic recycling.

Altogether, these studies suggest that Rnd2 and Rnd3 promote cortical neuron migration by distinct mechanisms that may involve selective interactions with different members of the plexin family of transmembrane receptors in different subcellular compartments.

## Concluding remarks

In the last decade, the introduction and constant refinement of new technologies, such as *in utero* electroporation of the murine embryonic cerebral cortex, have greatly advanced our understanding of the molecular pathways operating in migrating neurons (Loturco et al., 2009). Through the control of cytoskeleton remodeling, Rho and Rnd proteins have been shown to play crucial roles during neuronal migration in the developing cortex. However, whereas the cellular and molecular functions of Rnd proteins have been thoroughly described in cortical projection neuron development, very little is known about their role in tangentially migrating cortical interneurons. This would be a fertile territory for future research. Nonetheless, the critical function of Rnd proteins in the control of neuronal migration has been further highlighted by a recent study showing the requirement of Rnd3 for the tangential migration of newborn olfactory neurons from the SVZ to the olfactory bulb in the post-natal brain (Ballester-Lurbe et al., 2014).

*Rnd2* and *Rnd3* expression in the cortex is under the transcriptional control of the proneural factors Neurog2 and Ascl1, respectively. These factors are well known master regulators of neuronal differentiation and activate a transcriptional program of neurogenesis in neural progenitors (Bertrand et al., 2002). Since Rnd proteins also control other aspects of cortical development, such as progenitor proliferation and neurite extension, it is possible that different transcriptional factors exclusively regulate the expression of different Rnd members to couple specific neuronal migration phases with other neurogenic events.

At the molecular level, Rnd2 and Rnd3 control distinct steps of radial migration, by interfering with RhoA activity in different subcellular compartments. The bHLH transcriptional factors Ascl1 and Neurog2 induce the expression of Rnd proteins as a strategy to repress RhoA during radial migration (Hand et al., 2005; Pacary et al., 2011). A recent model proposes that the bHLH-Rnd pathways are responsible to maintain a low level of background RhoA activity, which is essential to promote neuronal migration, but at the same time RhoA activation may still be required for example downstream of plexin receptors to stimulate actin-based contractility in defined compartments of migrating neurons (Govek et al., 2011; Azzarelli et al., 2014). Indeed, RhoA downstream effectors, such as myosinIIB and mDia1/3, have been found enriched in the proximal region of the leading process and at the cell rear, just before nucleokinesis (Tsai et al., 2007; Solecki et al., 2009; Shinohara et al., 2012). Therefore, Rnd proteins finely orchestrate RhoA levels in migrating neurons, by directing its inactivation to specific subcellular compartments and by being also involved in the signaling that promotes its activation, as in the case of Rnd3.

These studies performed in neuronal cells will contribute to a better understanding of the regulatory function of Rnd proteins in the migration of other cell types. Indeed, Rnd proteins, especially Rnd3, have been shown to also control the migration of non-neuronal cell types, such as epithelial cells (Guasch et al., 1998) or cancer cells (Riou et al., 2010). Rnd3 seems to regulate cancer cell invasion mainly through its effects on RhoA/ROCK activity. However, the specific contribution of Rnd3 to cancer cell invasion is controversial, since it has been shown to both promote and inhibit invasion (Gadea et al., 2007; Klein and Aplin, 2009), suggesting that Rnd3 may act via more than one molecular mechanism (Riou et al., 2010). Further investigation of Rnd functions in cancer cell migration will thus be crucial to a better understanding of the metastatic and invasive behavior of cancer cells.

In conclusion, it is becoming evident that Rnd proteins play important roles in cell migration during mammalian cortical development and in particular, considering their relatively recent evolution, it is possible that they might be involved in mechanisms of brain developmental and neuronal plasticity that are exclusive to vertebrate organisms.

### Conflict of interest statement

The authors declare that the research was conducted in the absence of any commercial or financial relationships that could be construed as a potential conflict of interest.
